# Tuned to language: the influence of musicality on phonaesthetic judgments

**DOI:** 10.3389/fpsyg.2026.1668062

**Published:** 2026-05-25

**Authors:** Lukas Nemestothy, Susanne Maria Reiterer

**Affiliations:** 1 Department of Linguistics, University of Vienna, Vienna, Austria; 2 Vienna Doctoral School Cognition, Behavior and Neuroscience, Vienna, Austria; 3 Vienna Cognitive Science Hub, Vienna, Austria

**Keywords:** aesthetic judgment, eroticity, musicality, musilanguage, phonaesthetics, phonetic perception, singers, singing abilities

## Abstract

**Introduction:**

Aesthetic perception is a cross-modal phenomenon that integrates sensory, emotional, and cognitive processes. While traditionally focused on visual arts and music, recent research has extended this framework to phonaesthetics, which explores affective judgments of language sounds. This study investigates the intersection of musicality and phonaesthetic judgments, particularly focusing on the dimension of eros (eroticity) in language perception. Musicality, encompassing sensitivity to pitch, rhythm, and timbre, has been linked to language aptitude, prosodic sensitivity, and emotional processing in speech. However, its role in shaping affective-aesthetic evaluations of language remains underexplored.

**Methods:**

The study involved 203 participants, who were asked to give affective-aesthetic judgements of 24 languages (48 recordings). Musicality was assessed through self-reported general aptitude, singing skills, and instrumental skills. Familiarity with the languages was controlled to isolate the effects of musicality.

**Results:**

Results revealed that general musicality showed a trend in predicting eros ratings, but singing skills emerged as a significant predictor. Participants with higher singing abilities rated languages as more erotic, suggesting a shared vocal and prosodic foundation between singing and speech. Instrumental skills, in contrast, did not significantly influence eros ratings. Interestingly, the effect of singing skills on eros ratings was higher than the effect of familiarity, which was assumed to play a bigger role due to the mere-exposure effect.

**Discussion:**

The findings highlight the nuanced role of musicality in language perception, with singing skills offering a unique lens into the affective-aesthetic resonance of speech. This supports the notion of a vocal aptitude that bridges music and language, aligning with theories of a musilinguistic continuum. Additionally, the study suggests that singers may possess refined auditory and proprioceptive awareness, enabling more nuanced evaluations of unfamiliar languages. The results also raise questions about the influence of “exoticity”, as the sample consisted of rarer languages. This research contributes to interdisciplinary discussions on the interplay between music, language, and aesthetics, emphasizing the need for further exploration of sub-dimensions of musicality, or creativity in general. By bridging linguistics, musicology, and aesthetic psychology, the study opens new avenues for understanding how musicality shapes the emotional processing of language.

## 1 Introduction

Aesthetic perception is a deeply cross-modal phenomenon, engaging the senses, emotions, and cognition in the evaluation of experiences. While the empirical study of aesthetics has traditionally centered on the visual arts and music (Leder and Nadal, 2014; Scruton, 1997; Zeki, 1999) a recent trend has begun to extend this framework to linguistics, particularly through the lens of phonaesthetics, investigating the mechanisms behind the “beauty of language sound”. It builds upon the well-established field of linguistic iconicity or sound-symbolism which investigates the perceptual similarities between speech sound and concept meaning, juxtaposing the arbitrariness principle in certain domains. This debate dates back to Greek antiquity and to Plato's Cratylus Dialogue—where the relationship between sound and meaning are debated from both perspectives: iconicity vs. arbitrariness. Phonaesthetic research—slightly different to the field of iconicity *per se*—focuses on the listeners affective judgements of certain language features or languages as a whole (Anikin et al., 2023; Kogan and Reiterer, 2021; Mooshammer et al., 2024; Nemestothy et al., 2025; Reiterer et al., 2020). Beyond visual art, aesthetic responses are not limited to music or language as separate domains but can also be elicited by the human voice itself. Singing represents a particularly salient case, as it integrates linguistic and musical structures within a single expressive medium (Popescu et al., 2025). From evolutionary perspectives, language and music are often theorized to share roots in a proto-communicative vocal system in which rhythmic and melodic vocal displays served social bonding and mate attraction (Brown, 2017; Fitch, 2010, 2017; Mithen, 2009; Popescu and Fitch, 2025; Worden, 2022). This perspective predicts that speech sounds can carry affective value even when lexical meaning is unavailable.

Aesthetic responses to language sound are influenced by linguistic features, socio-cultural associations, and the listener's disposition. While work by Winkler et al. (2023) has opened this discussion on individual differences by relating language judgments to personality dimensions with the OCEAN model, other personal differences are still underinvestigated. Importantly, phonaesthetic judgments need not be limited to “beauty”: in this line of work, eros has been proposed as a distinct affective-aesthetic dimension that captures attraction to language sound (Kogan and Reiterer, 2021; Reiterer et al., 2020).

Musicality, broadly defined, encompasses perceptual and cognitive sensitivity to pitch, rhythm, and timbre, as well as behavioral engagement with musical performance and appreciation (Fitch, 2006; Honing, 2019). It is not limited to professional musicianship, even non-musicians vary significantly in their subjective musical aptitude and experience with music (Müllensiefen et al., 2014). These individual differences have been shown to influence language aptitude (Swaminathan and Schellenberg, 2020; Turker and Reiterer, 2021), prosodic sensitivity, and even emotional processing in speech (Christiner and Reiterer, 2013). In previous research (Reiterer et al., 2020; Kogan and Reiterer, 2021) hints were found toward a possible link between musical ear/musicality, plurilingualism and the phonaesthetic appreciation of languages. This raises a compelling question for aesthetics research: can musicality also shape how we perceive language? In the present study, we build on this framework by asking whether individual differences in musicality relate to phonaesthetic evaluations of spoken languages, with a particular focus on eros.

### 1.1 Musicality and aesthetic sensitivity

From an evolutionary standpoint, music and language are believed to have co-evolved from a common proto-communicative system, as proposed by Mithen (2009). In his hypothesis, Mithen argues that early hominins communicated through a pre-linguistic system that was holistic, musical, mimetic, and multimodal, long before the emergence of syntax. This mode of communication, heavily reliant on prosody, pitch, timbre, and rhythm, would have been crucial for emotional bonding, social cohesion, and mate attraction. As a result, humans may be biologically predisposed to respond to acoustic features in both speech and music in ways that are emotionally charged. Such a predisposition would help explain why some language sounds are consistently perceived as more beautiful or erotic, even in the absence of semantic understanding.

Cognitive and neuroscientific findings support this evolutionary linkage. Musical and linguistic processing share overlapping neural substrates, particularly in the auditory cortex, inferior parietal lobule, and inferior frontal gyrus—regions associated with both music and speech perception (Patel, 2007; Turker and Reiterer, 2021). Functional and structural imaging studies suggest that individuals with heightened musicality, especially those with advanced singing abilities, show increased gray matter volume and connectivity in these regions, which correlates with superior phonetic imitation, speech perception, and even foreign-language pronunciation. This link has led to the proposal of musicality as a key component of the “cognitive starter kit” for language learning (Turker and Reiterer, 2021).

The OPERA hypothesis (Patel, 2011, 2014) provides a compelling framework for understanding how musical training can influence speech processing through mechanisms of neural plasticity. The hypothesis states, since music and speech share cognitive processing mechanisms, and music places higher demands on these mechanisms than speech, musical training can enhance speech processing. This enhancement is driven by five key conditions: Overlap, Precision, Emotion, Repetition, and Attention. Of relevance to affective-aesthetic speech processing is the “E” component—Emotion. Patel (2014) emphasizes that music is deeply intertwined with emotional processing, often eliciting strong positive emotions and dopamine release, which are known to promote neural plasticity. Engaging with music creates a highly rewarding activity for the brain. This emotional engagement, combined with the persistent associations formed through long-term musical training, facilitates lasting neuroplastic changes. These changes are of importance to speech processing, which could further influence the sensibility to the emotional perception of speech (e.g., sensitivity to emotional prosody and vocal-affective cues). Koelsch (2015) articulates a principled framework in which music evokes emotion through mechanisms such as expectancy, resonance, memory, and social cognition, and relates these mechanisms to neural activity in affective and reward systems, supported by neural activation in core emotional and reward structures like the amygdala, hippocampus, and ventral striatum. This underscores how musical experience does not merely reflect emotion but actively engages and modulates the brain's affective systems.

Musicality's influence extends beyond learning to low-level perceptual strategies. For instance, Cui and Kuang (2019) showed that individuals with higher musical aptitude—regardless of language background—rely more consistently on fundamental frequency (*F*0) in pitch discrimination tasks, demonstrating a more refined auditory resolution. In contrast, those with lower musicality relied on coarser spectral cues. These findings suggest that musicality may sharpen acoustic parsing abilities, which could extend into aesthetic evaluations of speech—particularly for sound dimensions like melodicity, rhythm, and timbre, which often underlie judgments of beauty in language.

Nardo and Reiterer (2009) further argue that musicality reflects a general auditory aptitude—with singing skills and rhythm perception especially predictive of phonetic language aptitude. Their findings show that musical sensitivity is not limited to musical contexts but generalizes to speech-based tasks, notably in pronunciation and accent imitation. These findings suggest that individuals with higher musicality may be more attuned to the acoustic and aesthetic contours of language, shaping how they perceive it. Li et al. (2024) found that individual differences in musical perception, especially in melody processing for Catalan speakers and accent perception for Chinese speakers, strongly predicted performance on speech imitation tasks, independently of working memory capacity. These findings support the idea that musical aptitude plays a direct role in how individuals perceive and learn novel spoken forms.

### 1.2 Creativity without a creator

Empirical Aesthetics, as discussed in the previous section, focuses mainly on the visual arts (Leder and Nadal, 2014; Scruton, 1997; Zeki, 1999). Investigating language as a subject in empirical aesthetics might change some theoretical aspects of the discussion. An important concept of aesthetics is the interplay of creator and consumer—since pieces of art are mostly on purpose imbued with aesthetic features that possibly result in an aesthetic response of the consumer. The creativity-aesthetics cycle (Brown, 2022, 2024) displays the influences of both sides—production and perception. This cycle works well in some sub-parts of phonaesthetic research but might need adaptation for the investigation of natural languages. In research on constructed languages (Mooshammer et al., 2024) we clearly have a creator of a language, and the creator aims, in most cases, to include features that people find attractive in their con-lang. For example, the Elven languages of Tolkien, Sindarin and Quenya, aimed specifically to include aesthetic features and are perceived as noble, good and attractive (Mooshammer et al., 2024). Others, such as Klingon, aim to and were perceived as sounding unpleasant, evil, and harsh.

Darwin's original theory of sexual selection was explicitly aesthetic in nature, framing mate choice as grounded in a “taste for the beautiful” rather than exclusively in utilitarian indicators of fitness. As emphasized by Prum (2012), this aesthetic dimension was not a metaphorical flourish but a core theoretical claim: Darwin explicitly conceived mate preferences as an aesthetic faculty capable of evolving independently of direct adaptive benefits. Within this perspective, aesthetic traits and aesthetic preferences are understood as coevolving systems, shaped through reciprocal interactions between signal production and cognitive evaluation (Prum, 2012). Such coevolutionary dynamics resonate with contemporary accounts of the shared evolutionary origins of music and language, in which vocal displays function as communicative acts that are evaluated not solely for meaning or efficiency, but for their affective and aesthetic impact. From this view, language and music can be conceived as part of a broader aesthetic-communicative continuum, in which the perception of something as arousing, attractive, romantic or erotic (eros scale) are central evolutionary drivers rather than by-products.

Investigating phonaesthetics on a word/semantic level often focuses on how sound patterns contribute to stylistic effects and meaning in specific lexical items. Crystal (1995) investigated the aesthetics of English words and found that there need not always be an overlap of semantic beauty and phonetic beauty. Therefore, a poet/author/journalist might aim to include many phonaesthetically beautiful words in a text to elicit aesthetic judgments by the consumers. This can be understood as one route by which producers strategically shape aesthetic input for recipients, thereby closing the production-perception loop described in the creativity-aesthetic cycle (Brown, 2024).

**Figure 1:**
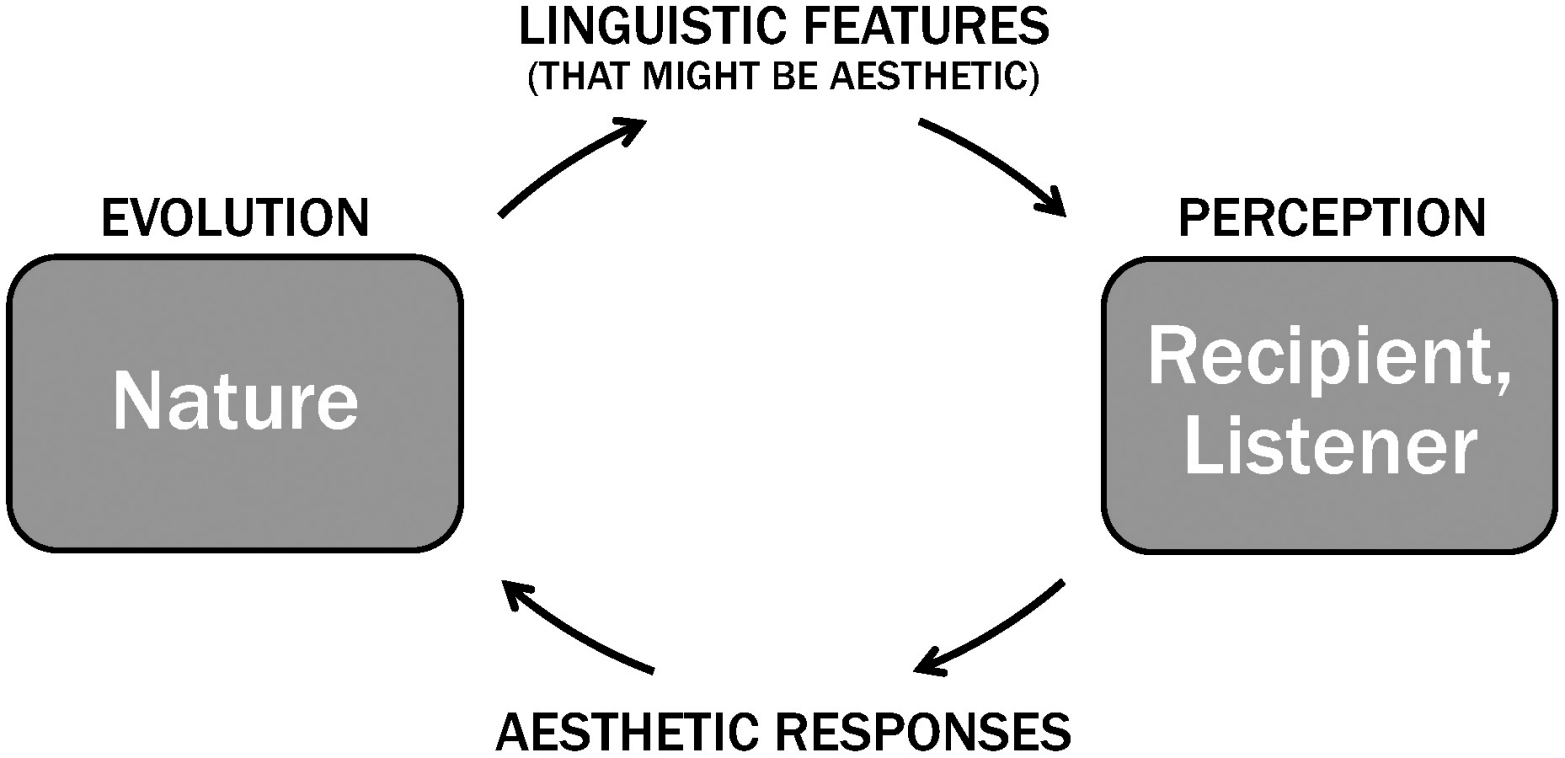
The phonaesthetic cycle: Adapted from the creativity/aesthetics cycle from Brown (2024, p. 3; Figure 2). The chart depicts the nature of language, formed by evolutionary and historical processes (influenced by natural and sexual selection). Languages have specific features of which some are of aesthetic value. The recipient or listener perceives the language and elicits aesthetic responses which then influence the language *per se* again.

The aim of the present study is to investigate natural languages as a whole, to preserve as much of the authentic aesthetic-acoustic speech pleasure as possible—in contrast to word-level studies or experimentally manipulated micro-stimuli (e.g., single words or crafted items; Crystal, 1995). In this investigation, we cannot clearly posit a “creator” that aims to include aesthetic features in a language. These processes might better be explained by the evolution of language (Fitch, 2010, 2017) and certain mechanics of mating calls and sexual selection (Worden, 2022) that overlap with our understanding of the mechanics behind human language. Rather than assuming that languages “aim” to be aesthetically pleasing, we treat phonaesthetic value as emerging in interaction: speakers produce vocal signals under biological and sociohistorical constraints, and listeners evaluate these signals, which can feed back into patterns of use, prestige, accommodation, and—over longer timescales—language change. However, the “creativity” aspect is more opaque and a rather long process. Figure 1 shows an adapted creativity-aesthetics cycle for the means of investigating the aesthetics of natural languages. There is no clear process of “production” in the sense of the creation of language if we look at the level of whole language systems (from a macro perspective). Each individual speaker chooses to produce a language more or less creatively—but this is not the phenomenon we investigate. The creator here is simply put “nature” (i.e., evolutionary and sociohistorical processes acting over time), or nature over time (history), which is taken from evolutionary theories on language. However, it can also be argued that modern languages are subject to quick changes if we think of the dynamics of youth language, loanwords, migration and influences of cultural phenomena. In the present study we chose to look at it on a macro-level and therefore introduce nature as the “creative” component of language, however there might also be good arguments to introduce “society” or other creative subjects in order to portray different approaches to the complex concept of human language.

The present study aims to approach the phonaesthetic interplay from a macro perspective. The suggested adapted creativity-aesthetics cycle (Figure 1) can be read as follows: a language comes with a certain set of linguistic features. Since languages are not purely created for aesthetic pleasure—and other functions, such as intelligibility or information processing, might be of greater importance—only some of these features might be considered aesthetic. The recipient or the “consumer” of a language perceives this language and can form aesthetic responses—for example immediate affective impressions (e.g., liking, arousal, eros), perceived social qualities (e.g., status), and approach/avoidance tendencies that can translate into downstream social evaluations and communicative choices. These judgements are, similarly to the arts, subject to change. These aesthetic responses change how a language is used and contribute to the evolution of languages, especially if we consider that languages are used in mating calls, which accounts for theories on sexual selection and its influence on language development (Worden, 2022). Additionally, according to Jakobson (2019) the aesthetic function is one of the core features of language. The phonaesthetic cycle remains largely close to the creativity-aesthetics cycle (Brown, 2024) but is distinct in the production phase, since we have—so to say—all of humanity as the producers, or all the speakers of the respective language.

### 1.3 Voice in the musilinguistic continuum

The debates around the Proto-Musilanguage also call for a (re-)unification of language and music (Brown, 1999). Sachs (1943) calls for two origins of music, the melogenic type which is derived from melody and the logogenic which is derived from language. These distinctions depict a continuum of vocal productions. Ranging from speech to logogenic music, to melogenic music with lyrics and to purely melogenic music (Brown, 2017). To give examples the dimensions range from a politician's speech, to rap, then pop music, and finally scat-singing. Most of the forms of vocal production still rely on lexical features and have in some way linguistic meaning imbued in them, whether or not this is comprehensible. Only vocable singing, as a pure melogenic form, is behind the “linguistic wall” (Brown, 2017) which shows purely non-lexical functions. The linguistic wall creates a divide in the musilinguistic continuum and illustrates the connection between (most) singing performances and typical language use. Instrumental music is behind the figurative “linguistic wall,” the exception being instrumental speech surrogates.

The musilinguistic continuum shows that language and music, although often treated as distinct fields, overlap to a great degree. Recent empirical work further substantiates this integrative perspective. Investigating late-Romantic choral songs, Popescu et al. (2025) demonstrate that tonal (musical) and semantic (lyrical) tension interact in complex ways during naturalistic listening. Using continuous tension ratings and partial information decomposition, they show that while musical tension tends to dominate holistic affective appraisal, lyrics contribute both redundantly and synergistically to the overall experience. Crucially, their findings suggest that the integration of music and language in vocal music is not merely additive but can exhibit synergistic effects, supporting the notion that songs are “greater than the sum of their parts”. This empirical demonstration of cross-domain integration provides a contemporary operationalization of the musilinguistic continuum and highlights how vocal production uniquely binds linguistic and musical structures within a shared affective trajectory.

Considering the concept of the “linguistic wall” speech lies closer to songs with lyrics than to instrumental music, which lies on another dimension of production and has almost always no lexical features (the exception being speech surrogates, like talking drums where the distinction between voice and instrument is blurry since the imitation of the human voice is key; Brown, 2017). Therefore, this framework motivates a distinction between vocal and non-vocal musical expertise when predicting phonaesthetic judgments: because singing engages the same vocal apparatus as speech, singing skills are expected to relate more strongly than instrumental skills to affective-aesthetic ratings of spoken languages, particularly in the eros dimension.

A central implication of this continuum is that speech perception is not purely auditory: it is intimately linked to the motor system that produces vocal sound. Listeners can (implicitly) map perceived speech sounds onto articulatory possibilities, which makes voice perception an embodied experience. In embodied cognition terms, this mapping can be framed as a form of proprioceptive and kinaesthetic anticipation—recently discussed as “proprioceptive resonance” (Sanna, 2025)—and it may be particularly pronounced in individuals with refined vocal control (Patel, 2011, 2014; Christiner and Reiterer, 2013, 2015).

### 1.4 Eros as an embodied affective-aesthetic dimension

Across phonaesthetic dimensions, eros is uniquely suited to connect aesthetic appraisal to theories of vocal signaling: it captures an attraction- or approach-related response to language sound that is plausibly rooted in the evolutionary perspectives (Worden, 2022; Fitch, 2010, 2017). In this sense, eros can be treated as an affective-aesthetic outcome that is closer to motivational relevance than general pleasantness.

Crucially, an aesthetic framework of sexual selection highlights that certain affective responses are more tightly coupled to mate choice than others. Prum (2012) argues that aesthetic evolution is fundamentally grounded in sensory and cognitive evaluation leading to preference and choice, rather than in detached assessment of encoded information. From this perspective, aesthetic responses are intrinsically motivational: they govern attraction, approach, and selection.

Unlike sight, sound is inherently intrusive; it does not remain at a distance but “vibrates” within the listener's ear. This acoustic immediacy can create a sense of simulated intimacy. Whispering, in particular, has been shown to trigger Autonomous Sensory Meridian Response (ASMR) in many listeners. Although ASMR is not necessarily sexual, it involves a tingling sensation associated with activation in brain regions implicated in pleasure and reward processing. More generally, when a voice is perceived as attractive or rewarding, it engages the mesolimbic reward system, the same circuitry involved in other pleasurable experiences such as food consumption or music listening. For further discussion of how vocal attractiveness influences behavior and decision-making, see Shang and Zhang (2025), who describe a “beauty premium” effect in vocal communication.

In this sense, eros can be understood as a particularly relevant aesthetic dimension, as it directly indexes the motivational and embodied components of attraction that are central to mate choice. Eros captures the extent to which a stimulus elicits approach-related affect and experiential engagement. When applied to spoken language, eros thus reflects not merely a preference for certain sound structures, but an affective response rooted in the perception of human vocal displays as biologically and socially meaningful signals (Prum, 2012). Recent work on synthetic voice design illustrates how specific acoustic features such as pitch and breathiness systematically shape gendered perceptions. Lozo (2026) demonstrates that higher pitch and increased breathiness in artificial female voices are associated with traditional, affiliative femininity, whereas lower pitch and creaky phonation index a more professional or authoritative femininity. These findings underscore that even subtle vocal parameters can modulate affective responses to speech and might illustrate differences in perceived attraction between genders.

Empirically, eros has been established as a separable judgment dimension in phonaesthetic work on European languages (Kogan and Reiterer, 2021; Reiterer et al., 2020). Theoretically, eros is expected to depend not only on familiarity or socio-cultural stereotypes, but also on acoustic–prosodic cues (e.g., timbre, pitch dynamics, rhythm) and on the listener's sensitivity to these cues—especially when semantic comprehension is absent.

This motivates the present focus on singing skills and whether they enhance auditory precision, affective engagement, and auditory-motor coupling (Patel, 2011, 2014; Koelsch, 2015; Christiner and Reiterer, 2013, 2015), it may also amplify embodied attraction responses to speech sounds. Importantly, our models therefore control for familiarity (Zajonc, 1968), allowing us to test whether vocal expertise contributes beyond mere exposure.

Building on the idea that singers may develop heightened sensitivity to vocal timbre and prosody, we explore whether vocal expertise is associated with stronger eros judgments particularly for less familiar languages. This hypothesis is consistent with the notion that refined control of the speech apparatus supports richer embodied simulation during listening (Sanna, 2025; Christiner and Reiterer, 2013, 2015).

## 2 Aims and hypotheses

The present study investigates whether self-perceived musicality predicts affective-aesthetic evaluations of spoken language, with a focus on the dimension of eros. Ratings of perceived eros are of special interest due to the arguments of sexual selection in context of the evolution of language. A compiled dataset is used in which each participant rates 24 languages (the procedure is explained in: Winkler et al., 2023; the same parent dataset is also used in: Kogan et al., under review; the present study is a secondary analysis focusing on musicality, whereas the other study investigates a wide range of mainly phonetic predictors with Bayesian models). The dataset includes information on individual musicality traits and their relationship to affective ratings. The project offers a novel investigation into how musical self-concept modulates the aesthetic perception of language. The study focuses on individual differences in musicality and how these relate to cross-linguistic variation in aesthetic judgments while controlling for familiarity, a known strong predictor of affective ratings (Zajonc, 1968: mere exposure effect).

**Primary hypothesis:**

Self-perceived general musicality (including musicality, singing skills, and instrumental skills) positively predicts affective-aesthetic (eros) ratings of spoken languages, when controlling for familiarity.

We expect this positive association because musicality—especially vocal skill—enhances sensitivity to pitch, timbre, and prosodic expressivity, and musical engagement tends to heighten affective responsiveness to auditory stimuli. Together, these factors should increase the likelihood of stronger eros judgments even when familiarity is held constant. However, all models aiming to test the hypothesis are two-tailed, therefore the direction is merely stemming from the author's expected effect.

**Secondary hypothesis:**

Singing skills will be a stronger predictor of affective-aesthetic language ratings than instrumental skills, due to the shared vocal and prosodic features.

These hypotheses are designed to disentangle the global and language-specific effects of musicality on listeners' judgments. The primary hypothesis is formulated with a direction but is however anyway tested two-tailed. The study takes an exploratory-comparative approach to assess whether certain types of musical experience (e.g., vocal vs. instrumental) and certain languages elicit stronger or more consistent associations with perceived eros. An additional exploratory analysis will test whether the musicality-eros association varies across languages, indicating language-specific sensitivity to musical traits. Due to an expected overlap in the aesthetic-affective dimension of language ratings a *post-hoc* analysis of the beauty ratings were performed, in order to shed light on the differences between these dimensions.

## 3 Materials and methods

### 3.1 Participants

A total of 203 participants took part in the study, completing all components of the experimental protocol (compiled dataset, for the experimental procedure see Winkler et al., 2023). All participants completed a combination of listening and rating tasks, followed by a comprehensive background questionnaire assessing individual differences in linguistic exposure and musical self-perception.

The sample had a mean age of 32.35, with a standard deviation of 10.05. The youngest participant was 12 years old, the oldest 73. The sample was rather multilingual with a mean of 4.15 (SD +/– 2.03) languages spoken to some degree (“Please indicate the number of languages you know plus provide the European reference level and a gradual rating of your fluency”). In relation to their gender the participants were to the biggest part female (135, ~66.5%), with 65 participants (~32%) male and 3 participants that had a “non-binary” gender identity (~1.5%).

The participants named 34 different countries of origin and one stated “other” when asked for their birthplace. The majority of the participants originated from China (*n* = 61; ~30%), followed by Austria (*n* = 39; ~19%), Germany (*n* = 22; ~11%), the United States of America (*n* = 15; ~7%) and Slovenia (*n* = 11; ~5%). All the other birthplaces mentioned accounted for under 4% of the sample. Since this study is focused mostly on language the L1 (“native or first language”) of the participants was also asked for. The biggest group were Chinese speakers (*n* = 65; ~32%) with 10 being Chinese bilinguals (~5%), followed by German speakers (*n* = 59; ~29%), English speakers (*n* = 23; ~11%), Slovene (*N* = 11; ~5%), Italian (*n* = 7; ~3%), Spanish (*n* = 5; ~2.5%) and Russian (*n* = 5; ~2.5%). All the other first languages were named less than five times. In total 26 different first languages were named.

It is important to note that all the ratings of one's L1 (“native or first language”) or an L2 (any language a participant stated that they can speak at any level A1–C2) have been excluded case-wise. This means that only ratings of participants that did not speak the language on any level (from beginner to native speaker) were left in the dataset.

### 3.2 Procedure

Data collection was conducted online, allowing participants to complete the experiment remotely in a quiet environment of their choosing. The procedure started with a self-report questionnaire, which assessed biographic information, linguistic background and musicality.

Each participant was presented with auditory stimuli from 24 languages, selected to represent a wide range of European language families. The European languages were selected in order to ensure that we can have most of the recordings from native speakers present in our sound studio. All recordings were standardized in terms of length, speaker characteristics (younger female voices instructed to speak in a neutral friendly tone), and acoustic quality to ensure perceptual comparability across languages. The study included only female voices since Reiterer et al. (2020) found that female voices were generally preferred by both male and female participants. Each participant listened to 24 language recordings (consisting of two voice subsets—two female native speakers per language—resulting in 48 recordings in total) in a randomized order to reduce potential sequence effects. After each recording, participants were asked to rate the language on four dimensions: beauty, eros, status, and order. These measures were designed to capture both affective and social impressions. In the current study, only the affective-aesthetic dimension of eros is considered. After rating each stimulus, the participants were asked to guess which language they heard, they were asked both the language and the language family of the stimulus they just heard. This measurement was used to indicate their familiarity with the language.

### 3.3 Measurement instruments

#### 3.3.1 Independent measures

*Musicality:* Musicality was assessed through self-report as part of a larger questionnaire. Self-rated general musicality was assessed with the question, “How musical would you say you are?” on an 11-point Likert scale (0 = not at all musical, 10 = extremely musical).

*Singing Skills:* Participants rated their singing ability on an 11-point Likert scale.

*Instrument Status:* Participants indicated whether they played any musical instruments (yes/no) and, if applicable, reported the number of instruments played.

*Instrumental Skills:* Participants rated their skill level for the musical instrument they played best (0–10 scale).

*General Musicality:* A compound score of the individual sub-scores of musicality, singing skills and instrumental skills. This represents the mean value of the three sub-measurements per participants (0–10 scale).

Musicality was assessed using three self-report items capturing general musicality, singing skills, and instrumental skills, all measured on a 0–10 scale. As these items were repeated across rating trials, internal consistency was assessed at the participant level. The three items showed good internal consistency (Cronbach's α = 0.80) and were averaged to form a composite score (gMusicality). At the same time, item-level analyses indicated that the components were related but not redundant, motivating a second modeling step in which musicality was decomposed into its constituent facets to examine facet-specific effects. Although these skills were assessed with a self-assessment, previous studies (Coumel et al., 2019) found that results obtained through self-assessment were yielding comparable results to actual assessments with recordings being evaluated by singing experts. This holds true for a mixed cohort of layman and professional singers—since the self-estimation of professionals is different—the present study consists of a random sample consisting mostly of laymen.

#### 3.3.2 Covariate

*Familiarity:* Familiarity with each language was assessed through a recognition task in which participants guessed the language they had just heard or named a close relative. The participants were asked: What language is this?—in case of doubt, name the language family; What could be a close relative of this language?. This gave the participants the chance to guess a close relative. Responses were scored from 0 to 3:

0 = completely unfamiliar

1 = correct wider language family only

2 = correct closely related language (e.g., Spanish instead of Catalan)

3 = exact match

This measure aims to control the influence of familiarity on aesthetic evaluations. The “recognition score” serves as amore indirect assessment of familiarity, since asking straightforward for familiarity might result in a biased answer or pure guessing. This provides a robust measure of language exposure and helps to control perceptual biases that may stem from prior experience. This does not include any language proficiency, since if a participant had any proficiency in a language the ratings were excluded case-wise.

#### 3.3.3 Dependent measure

*Affective-Aesthetic Ratings (Eros):* After each recording, participants rated “How erotic do you find this language?” using a continuous slider (0–100). Higher scores indicated a stronger perceived erotic quality.

### 3.4 Data analyses

Analyses were conducted using R (Version 4.4.1; R Core Team, 2025) using the package lme4 (Bates et al., 2015). Data were modeled using linear mixed-effects models (LMMs) to account for the hierarchical structure of the data (multiple language ratings nested within participants).

Model 1: predicted eros ratings from general musicality and familiarity.
Formula Model 1:
  *eros* ~ *gMusicality* + *familiarity* + (1 | *id*) + (1 | *language*)
Model 2: predicted eros ratings from the three musicality subcomponents (general musicality, singing skills, instrumental skills) and familiarity.
Formula Model 2:
  *eros* ~ *musicality* + *instrumentSkills* + *singingSkills* +*familiarity* + (1 | *id*) + (1 | *language*)

The first model investigates the influences of a general musicality score (i.e., the mean musicality value of three sub-scores) and the second model focuses on the sub-dimensions (musicality, instrumental skills and singing skills). Random intercepts for participants and languages were included in both models to account for individual variation and language-specific baseline ratings. The data is hierarchically structured, since each participant rated all languages.

The models include random intercepts for participants and languages, specified as (1 | id) and (1 | language). The participant-level random intercept accounts for stable individual differences in overall rating tendencies, such that some participants may generally provide higher or lower ratings across stimuli. The language-level random intercept captures baseline differences between languages, independent of the predictors of interest, as some languages may elicit generally higher or lower ratings. This specification was chosen because the primary research question concerns variation in affective-aesthetic evaluations across languages while controlling repeated observations within participants.

Although each language was represented by two recordings, language was treated as the theoretically central grouping factor in the main analyses, because the focus lies on language-level evaluations rather than on idiosyncratic differences between individual recordings. The two recordings per language were used to reduce the influence of a single speaker realization and to increase stimulus robustness within each language category.

Model assumptions (normality of residuals, homoscedasticity) were checked and met. Familiarity was retained in all models as a covariate given its known influence on language ratings. Both unstandardized and standardized regression coefficients (β) with 95% confidence intervals were reported. Statistical significance was evaluated at *p* < 0.05.

## 4 Results

### 4.1 Descriptive statistics

#### 4.1.1 Participant flow

An initial sample of 227 participants completed the experimental protocol. Twenty four participants were excluded after checking their rating behavior on the four dimensions (eros, beauty, status, order). If a participant had in at least two of four dimensions a SD = 0 they were excluded listwise. After these exclusion criteria 203 participants remained in total. Ratings for any language that participants reported speaking (L1 or L2, first or second language, at any proficiency level) were excluded on a case-wise basis to avoid biases from language proficiency. After excluding these ratings case-wise 4,681 observations were used for the main analyses.

#### 4.1.2 Descriptive statistics

Table 1 presents the descriptive statistics for all variables. On average, participants rated the eroticity of the languages at *M* = 47.38 (SD = 21.41) on a 0–100 scale. The mean general musicality was *M* = 5.05 (SD = 2.28), with musicality averaging *M* = 6.22 (SD = 2.47), singing skills averaging *M* = 5.15 (SD = 2.64) and instrumental skills averaging *M* = 3.79 (SD = 2.98) on a 0–10 scale. Mean familiarity scores (recognition accuracy, 0–3 scale) were *M* = 0.80 (SD = 1.09).

**Table 1:** Descriptive statistics for all variables.

Variable	*M*	*SD*	Range
Musicality	6.22	2.47	0–10
Instrumental skills	3.79	2.98	0–10
Singing skills	5.15	2.64	0–10
General musicality	5.05	2.28	0–10
Eros ratings	47.38	21.41	0–100
Familiarity	0.80	1.09	0–3

M, mean; SD, standard deviation. Musicality variables were self-reported on 0–10 scales. Eros ratings were measured on a continuous 0–100 scale. The composite musicality index is the mean of the three musicality measures. *Familiarity* represents the recognition accuracy of the language stimulus (0, unfamiliar; 3, exact match).

#### 4.1.3 Zero-order correlations

**Figure 2:**
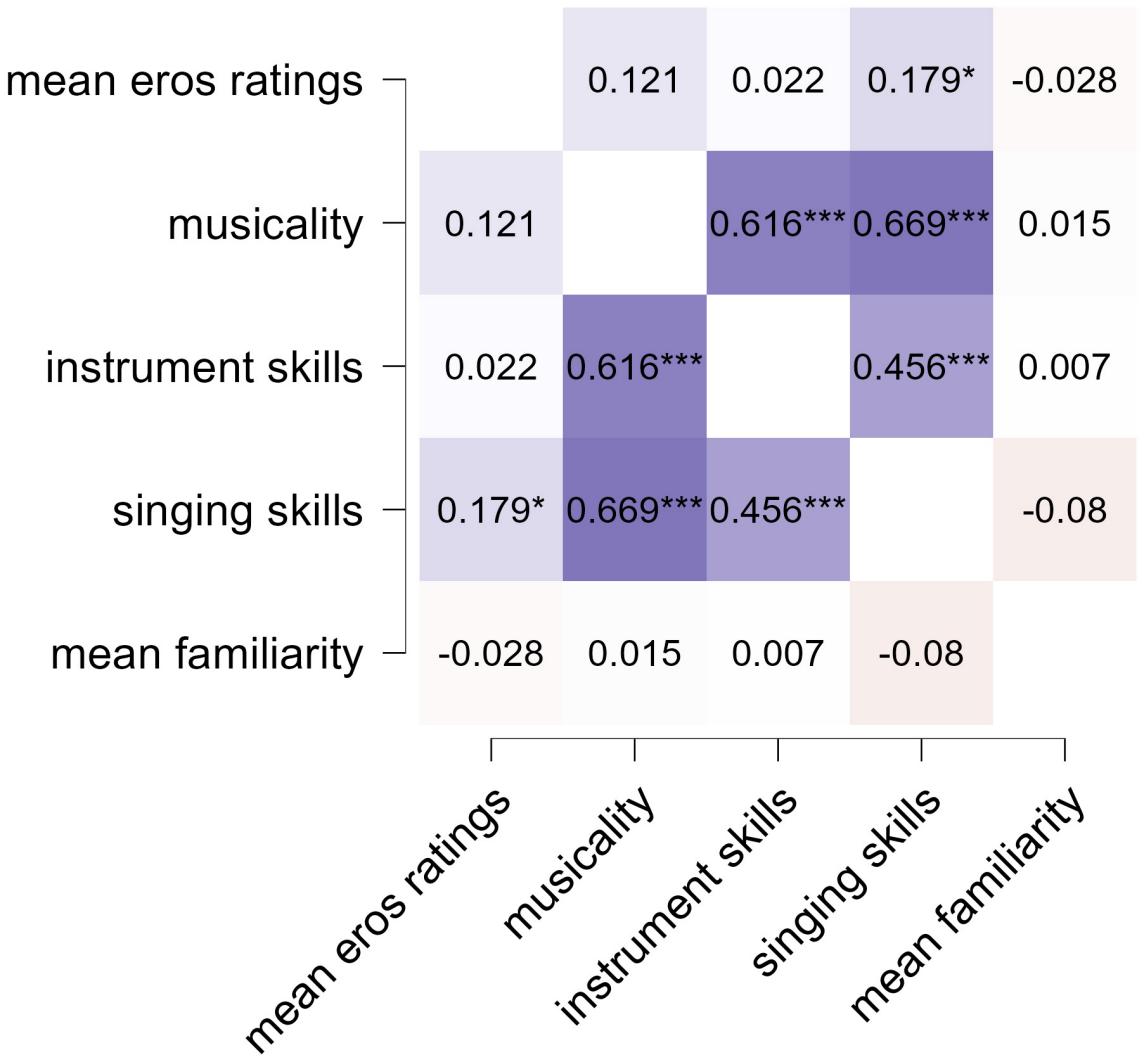
Zero-order correlations among study variables. Values represent Pearson correlation coefficients (*r*). Coefficients are based on the wide-format dataset, in which each participant contributed a single aggregated row. Mean eros ratings and mean familiarity were computed by averaging across all ratings provided by each participant to account for the hierarchical data structure. Darker shading indicates stronger associations. Asterisks denote statistical significance (*p* < 0.05, *p* < 0.01, *p* < 0.001).

Zero-order Pearson correlations were analyzed among all study variables using the wide-format dataset, in which each participant contributed a single row of data. Because the original dataset was hierarchical (multiple ratings per participant), both mean eros ratings and mean familiarity scores were calculated per participant before analysis. As shown in Figure 2, mean eros ratings correlated positively with singing skills, *r* = 0.18, *p* < 0.05, but were unrelated to general musicality, instrumental skills, or mean familiarity.

Musicality was strongly correlated with both instrumental skills, *r* = 0.62, *p* < 0.001, and singing skills, *r* = 0.67, *p* < 0.001. Instrumental skills and singing skills were also positively associated, *r* = 0.46, *p* < 0.001. Mean familiarity was not significantly correlated with any other study variable.

### 4.2 Hypothesis Testing

#### 4.2.1 Primary hypothesis—general musicality

The results of the first model and the primary hypothesis show the following fixed effects (Table 2):

**Table 2:** Fixed effects of the linear mixed model predicting eros ratings from general musicality and familiarity (model 1).

Predictor	*B*	SE	*t*	df	*p*
Intercept	42.43	2.38	17.84	222.02	< 0.001^*^
General musicality	0.71	0.40	1.77	201.24	0.078
Familiarity	1.72	0.27	6.27	4,631.85	< 0.001^*^

B, unstandardized regression coefficient; SE, standard error.

^*^indicate statistical significance at *p* < 0.05. Model includes random intercepts for participants and languages.

**Figure 3:**
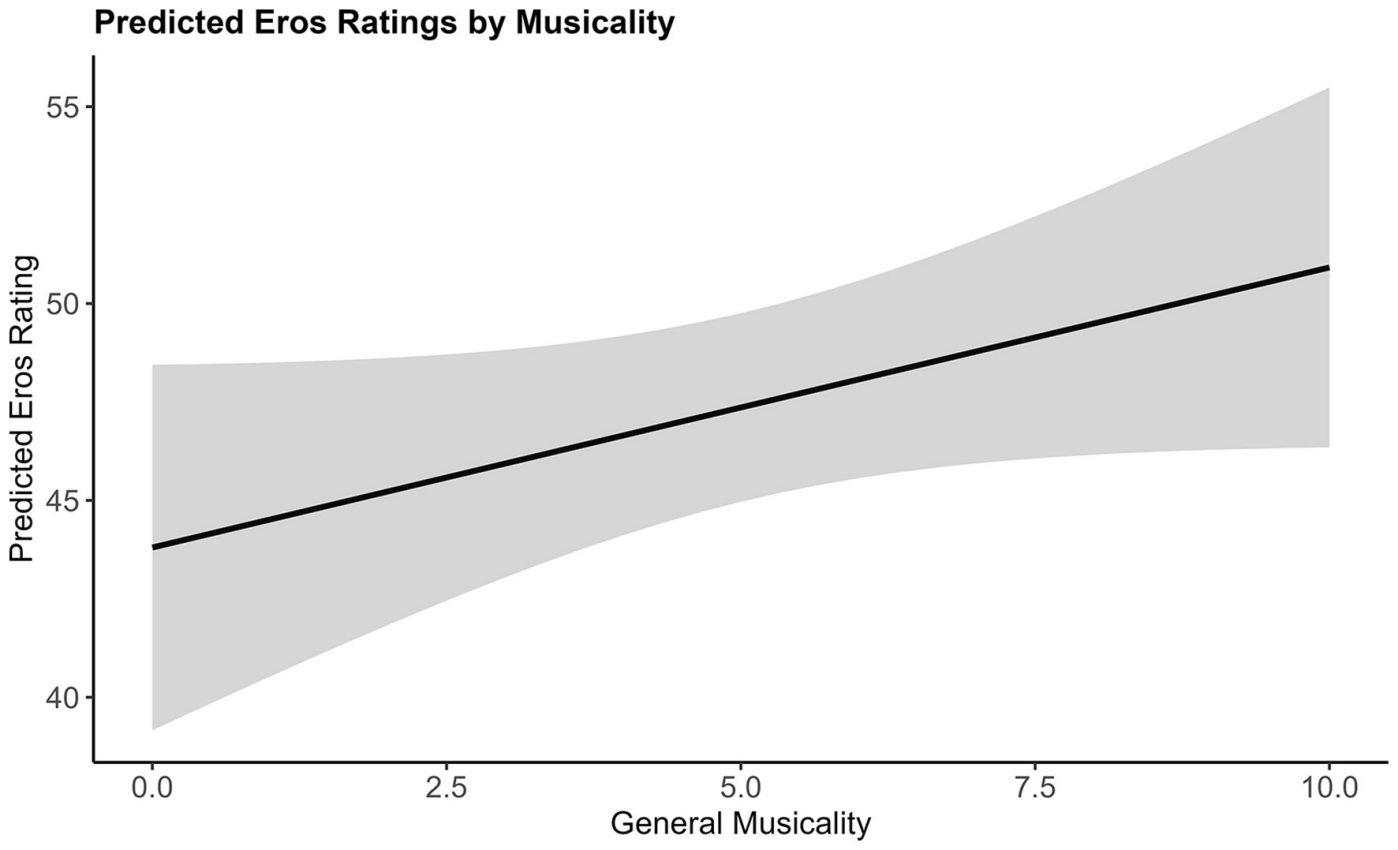
Visualization of the estimated relationship between general musicality (gMusicality) and predicted eros ratings, controlling for familiarity (recognition score). The regression line and a 95% confidence ribbon indicating the uncertainty of the estimate are shown. Although the effect of gMusicality is marginal (*p* ≈ 0.078), the figure illustrates a positive trend.

The result of the linear mixed model shows that familiarity with a language significantly influences the eros ratings (*p* < 0.001). These results were to be expected, and familiarity was kept in the model in order to control for effects related to familiarity. The general Musicality Index (consisting of the subdimensions analyzed in model 2) showed no significant influence on the eros ratings (*p* = 0.078). However, a trend might be assumed here since the *p* value is nearing the threshold of significance (Figure 3).

To assess the strength of the predictors in the mixed-effects model, standardized regression coefficients (β) were calculated. Self-reported musicality showed a small positive effect on eros ratings (β = 0.08, 95% CI [−0.01, 0.16]), though the confidence interval overlapped zero. Musicality shows a positive trend, suggesting more musical participants may give higher eros ratings, but it does not reach conventional significance. In contrast, familiarity with the language (measured via guessing accuracy) was a statistically robust predictor (β = 0.09, 95% CI [0.06, 0.12]), indicating that higher recognition scores were associated with higher eros ratings.

#### 4.2.2 Secondary hypothesis—musicality subcomponents

To disentangle the specific contributions of different facets of musicality on the perceived erotic appeal of languages, we fitted a linear mixed-effects model predicting *eros ratings* using three separate predictors: musicality, instrument skills, and singing skills, while controlling for familiarity. Random intercepts for both participants and languages were included to account for the nested data structure.

Multicollinearity among predictors was assessed using variance inflation factors (VIF). All predictors showed low VIF values (VIFs ≤ 2.33), indicating no problematic collinearity. This suggests that general musicality, singing skills, and instrumental skills capture overlapping but statistically distinguishable aspects of musical experience and can be included simultaneously in the model.

The second model was fitted to explore the sub-dimensions of musicality (Table 3). We can still see the expected result that familiarity with a language influences the ratings of a language. Two sub-dimensions, namely musicality and instrumental skills, did not show a significant influence on the ratings of eros. The singing skills, however, reach significance and therefore can be assumed to influence the aesthetic ratings on the eros dimension.

**Table 3:** Fixed effects of the linear mixed model predicting eros ratings from musicality subcomponents and familiarity (model 2).

Predictor	B	SE	*t*	df	*p*
Intercept	41.02	2.64	15.51	221.26	< 0.001^*^
Musicality	0.26	0.56	0.46	199.47	0.649
Instrumental skills	−0.43	0.39	−1.11	199.15	0.271
Singing skills	0.98	0.47	2.10	199.52	0.037^*^
Familiarity	1.73	0.27	6.30	4,630.93	< 0.001^*^

B, unstandardized regression coefficient; SE, standard error.

^*^indicate statistical significance at *p* < 0.05. Model includes random intercepts for participants and languages.

The model revealed a significant positive effect of singing skills on eros ratings (*p* = 0.037), indicating that individuals with higher self-rated singing skills tended to assign higher erotic ratings to the languages, regardless of their overall musicality or instrumental competence. In contrast, musicality (*p* = 0.649) and instrument skills (*p* = 0.271) did not significantly predict eros ratings, and their confidence intervals overlapped with zero. As expected, participants' familiarity with a language remained a robust predictor of eros ratings (*p* < 0.001). A direct Wald chi-square test comparing the singing-skills and instrumental-skills coefficients showed that singing skills were a significantly stronger predictor of eros ratings than instrumental skills (χ^2^(1) = 5.02, *p* = 0.025). The conclusion that singing-related self-perception was more strongly related to eros ratings than instrumental self-perception is therefore supported not only by the pattern of individual coefficients, but also by an explicit statistical comparison of the two effects.

**Figure 4:**
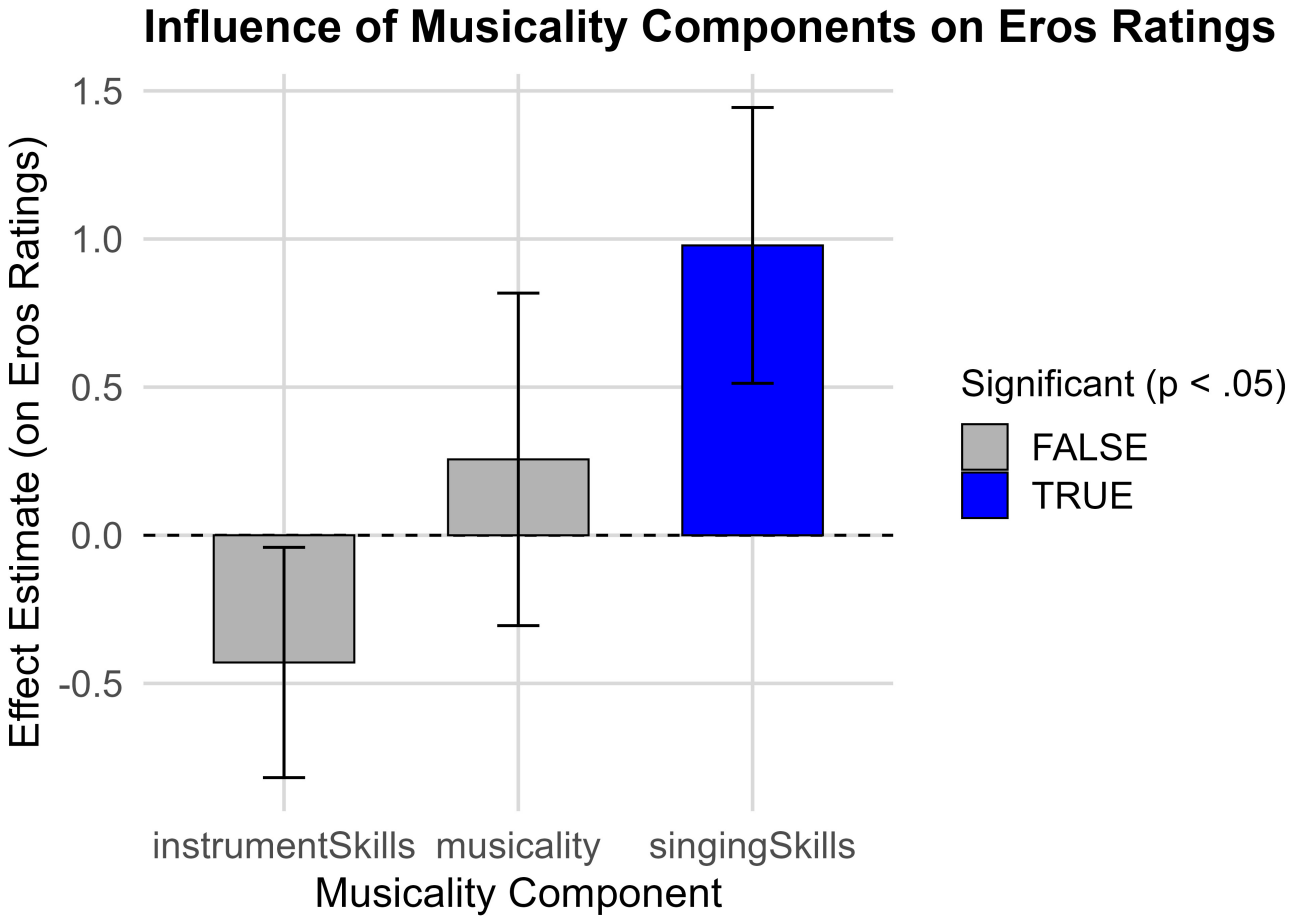
Effect estimates of different musicality subcomponents (instrument skills, musicality, and singing skills) on predicted eros ratings. Error bars represent 95% confidence intervals, with statistically significant effects (*p* < 0.05) highlighted in blue. The results suggest that among the musical subskills, singing skills show a significant positive association with eros ratings, while instrument skills and musicality do not show significant effects.

As illustrated in Figure 4, the results suggest that among the musical subskills, singing ability may play a particularly important role in modulating affective-aesthetic responses to languages on the eros dimension. The plot indicates the effect estimates (unstandardized effects) of the subcomponents.

The analysis of standardized effect sizes (β; Table 4) indicated that singing skills exert a small and statistically significant positive effect on eros ratings (β = 0.12, 95% CI [0.01, 0.23]). Interpreting the unstandardized regression coefficient, eros ratings increased by approximately one unit (*B* = 0.98) for every one-point increase in self-reported singing skills. Familiarity also exerts a small significant effect on eros ratings (β = 0.09, 95% CI [0.06, 0.12]), however the standardized effect of self-assessed singing skills is bigger.

**Table 4:** Standardized regression coefficients (β) for musicality subcomponents and familiarity predicting eros ratings (model 2).

Predictor	β	95% CI lower	95% CI upper
Intercept	0.00	−0.11	0.11
Musicality	0.03	−0.10	0.16
Instrumental skills	−0.06	−0.17	0.05
Singing skills	0.12	0.01	0.23
Familiarity	0.09	0.06	0.12

β, standardized regression coefficient; CI, confidence interval. Model includes random intercepts for participants and languages.

To assess robustness with respect to stimulus-level variance, the main model was re-estimated using a random intercept for individual voices (stimulus_set) instead of language. The voice-level random effect showed negligible variance, and fixed-effect estimates remained virtually unchanged, indicating that the observed associations are not driven by speaker-specific characteristics.

### 4.3 Exploratory analyses

An additional exploratory analysis was performed after discovering the influence of the singing skills on the eros ratings. A linear model (eros ~ singingSkills + familiarity) was fitted per language.

**Figure 5:**
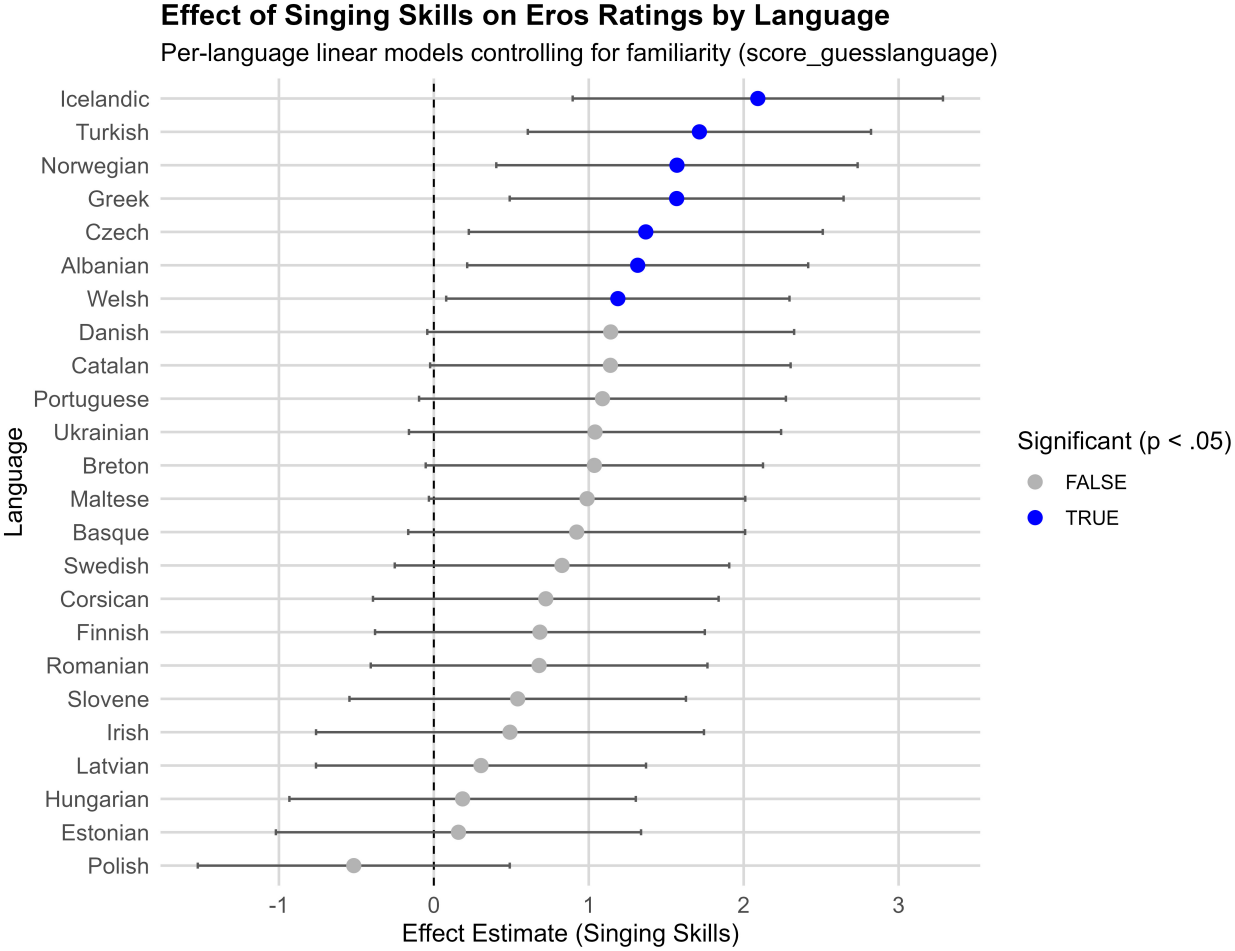
Language-specific effect estimates of singing skills on eros ratings, with 95% confidence intervals. Languages are ranked by the size of the effect. Uncorrected statistically significant effects (*p* < 0.05) are highlighted in blue. The figure highlights a general positive trend in most languages.

The exploratory analysis revealed that individual differences in singing ability significantly modulate eros ratings for a subset of languages. Uncorrected significant results (*p* < 0.05) were found in Icelandic, Turkish, Norwegian, Greek, Czech, Albanian and Welsh (Figure 5). In these languages participants with higher self-rated singing skills tended to provide significantly higher eros evaluations, when controlled for familiarity. The trend is overall positive for most languages (a positive shift), except for a few languages with no (significant) effect. After Benjamini–Hochberg correction for multiple testing across the 24 language-specific models, significant effects remained for Icelandic, Turkish, and Greek.

Languages where the effect was near zero (e.g., Latvian, Hungarian, Estonian and Polish) may fail to engage the same aesthetic sensitivities in skilled singers. Moreover, some languages showed positive trends that did not reach statistical significance, potentially due to variability in exposure.

**Figure 6:**
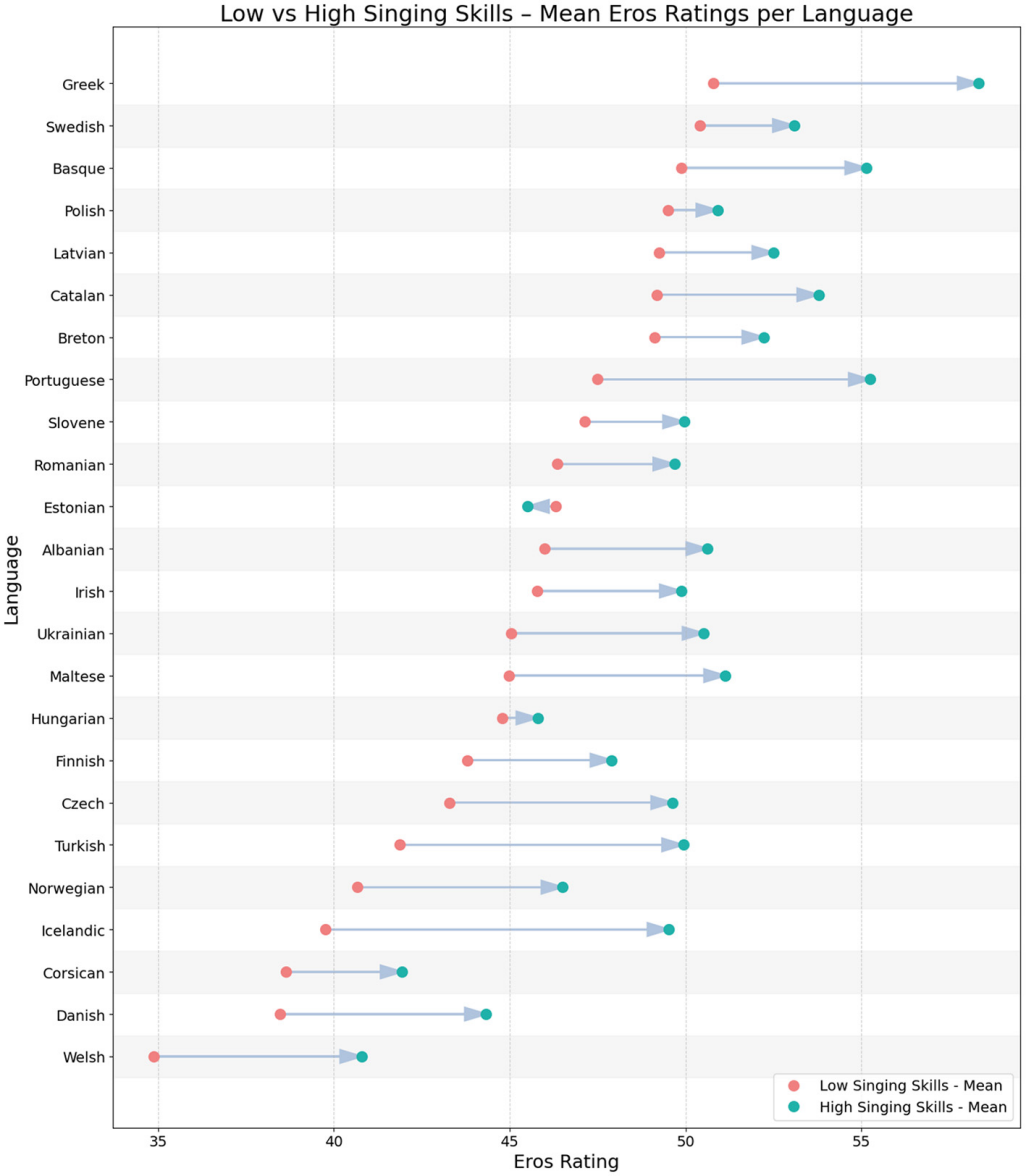
This figure indicates the mean values of each language for the groups (median split; high singing skills >5; low singing skills ≤ 5). The red dots, or the beginning of the arrow indicate the mean value of the eros ratings of the group with a low singing skills. The green dot, and the point of the arrow, indicates the mean value of the eros ratings of the singers (i.e., high singing skills).

It seems that there was not a language specific effect that would highlight a specific language family. The participants with higher singing skills rated the languages included in the study in general higher on the eros dimension. Figure 6 aims to illustrate this by comparing the eros ratings of the participants with lower singing skills (median split ≤ 5) to those of the higher singing skills (median split > 5).

In an additional exploratory analysis, age and gender were included as covariates in the final eros model. The pattern of results for the musicality-related predictors remained unchanged (Table 5): singing skills continued to show a significant positive association with eros ratings, whereas general musicality and instrumental skills did not. Familiarity remained a strong predictor of eros. Age showed no significant association with eros ratings. Gender showed a small effect, with female participants giving slightly lower eros ratings than male participants; however, this effect did not alter the musicality-related findings and is not interpreted further.

**Table 5:** Fixed effects of the linear mixed model predicting eros ratings from musicality subcomponents, familiarity, age and gender.

Predictor	*B*	SE	*t*	df	*p*
Intercept	43.79	2.92	14.99	221.72	< 0.001^*^
musicality	0.20	0.56	0.37	200.32	0.716
instrumental skills	−0.37	0.41	−0.92	199.88	0.361
singing skills	0.95	0.46	2.06	200.41	0.041^*^
familiarity	1.79	0.28	6.44	4,571.32	< 0.001^*^
age (z)	0.49	0.93	0.53	200.36	0.597
gender (female)	−3.81	1.93	−1.98	200.13	0.049^*^

B, unstandardized regression coefficient; SE, standard error.

^*^indicate statistical significance at *p* < 0.05.

Given that all stimuli consisted of female voices, the small gender difference may plausibly reflect differential baseline responses to speaker gender rather than a theoretically meaningful moderation of the musicality effect. The observed gender difference corresponds to a small effect (≈0.2 SD), with female participants giving slightly lower eros ratings than male participants. Given that all stimuli consisted of female voices, this difference could be interpreted as a weak hetero effect, in which female participants rated female voices lower than male participants.

### 4.4 *Post-hoc* assessment of beauty

Given the conceptual proximity between *eros* and *beauty* as affective-aesthetic dimensions, we conducted an exploratory *post-hoc* analysis to examine whether the observed musicality-related effects generalize beyond eros to judgments of beauty. Importantly, this analysis was not part of the original model and is therefore reported for hypothesis-generating purposes.

Using an identical structure to the final eros model, beauty ratings were predicted from musicality, singing skills, instrumental skills, and familiarity, with random intercepts for participants and languages (Table 6). In contrast to the eros models, none of the musicality-related predictors showed a significant association with beauty ratings. Neither general musicality nor singing or instrumental skills contributed meaningfully to the explanation of variance in beauty judgments. Instead, familiarity emerged as a strong and robust predictor, indicating that beauty ratings were primarily shaped by recognition-based factors rather than by individual differences in musical expertise.

**Table 6:** Fixed effects of the linear mixed model predicting beauty ratings from musicality subcomponents and familiarity (*post-hoc*).

Predictor	*B*	SE	*t*	df	*P*
Intercept	51.12	2.72	18.82	222.61	< 0.001 ^*^
Musicality	−0.16	0.59	−0.27	203.20	0.785
Instrumental skills	0.36	0.41	0.89	202.89	0.375
Singing skills	0.25	0.49	0.52	203.25	0.606
Familiarity	2.14	0.28	7.66	4574.38	< 0.001^*^

B, unstandardized regression coefficient; SE, standard error.

^*^indicate statistical significance at *p* < 0.05.

Crucially, multicollinearity diagnostics revealed low variance inflation factors, comparable to those observed in the eros models, ruling out collinearity as an explanation for the absence of musicality effects. Moreover, the lack of association was observed despite the large number of observations and the same statistical power that yielded a significant effect of singing skills on eros ratings.

Taken together, this dissociation suggests that musicality—particularly singing-related expertise—is not associated with a generalized tendency to evaluate language stimuli more positively. Rather, its influence appears to be specific to the eros dimension, supporting the view that eros reflects a distinct mode of affective response that is more closely linked to vocal expressivity and listener sensitivity to prosodic cues. In contrast, beauty judgments seem to rely more strongly on familiarity and recognition, pointing to partially separable evaluative processes underlying these two dimensions.

## 5 Discussion

The present study seeks to advance an interdisciplinary understanding of how musicality, especially singing ability, and language perception intersect within (phon)aesthetic experience. By examining individual differences in self-perceived musicality this study aims to uncover how listeners attune to the affective qualities of unknown languages, in the sense that they cannot speak or have learned them but could recognize some of the languages and name them (overall recognition rate of all languages was 14%). In focusing on the perceptual dimension of eros, the analysis sheds light on the emotional and aesthetic resonance of speech.

This research contributes to a growing body of work that views music and language not as isolated systems, but as interconnected aesthetic modalities governed by overlapping perceptual and cognitive resources. The concept of “the arts combined” serves as a foundation for this inquiry, highlighting that the different arts influence each other in various aspects of aesthetic perception. By investigating the aesthetic perception of language through the lens of individual self-assessed musicality, the study opens new avenues for interdisciplinary research at the intersection of linguistics, musicology, and aesthetic psychology.

The **primary hypothesis**, that self-perceived general musicality positively predicts affective-aesthetic (eros) ratings of spoken languages, when controlling for familiarity, cannot be statistically supported. General musicality showed a positive but nonsignificant trend.

The **secondary hypothesis**, whether singing skills will be a stronger predictor of affective-aesthetic language ratings than instrumental skills, is supported. Singing skills significantly predicted eros ratings, even after controlling for familiarity. Singing skills had a small effect on eros ratings, which is bigger than the effect of familiarity on eros ratings.

The **exploratory analysis** looked at how the effect of self-perceived musicality on language ratings varies across languages. Significant effects were observed across many, but not all, languages. However, no clear contrast between different language families could be supported. It seems that the participants with higher singing skills rated the languages included in the study in general higher on the eros dimension, with no clear preference for a language family.

Eros ratings appeared to reflect language-level affective evaluation rather than idiosyncratic properties of individual speakers, as no additional systematic variance attributable to speakers was detected beyond language-level differences.

### 5.1 Embodied vocal sensitivity and eros

The pattern of results is consistent with a domain-specific vocal mechanism: embodied engagement with the vocal tract (through singing) may sharpen auditory-motor sensitivity to speech, which in turn increases affective-approach activation during perception and is expressed as higher eros ratings. The present study aims to be a steppingstone to exploring the idea that perceiving speech can involve implicit simulation of articulatory gestures and their expressive potential.

The study investigates musicality both as a general score and in a separated form where different skills that compose musicality are highlighted. The primary investigation aims to look at the effect that the general self-assessed musicality has on the perception of eros (eroticity dimension) of languages' sound. This shows a (borderline-significant) trend that musicality in general influences the perceived eros in the sound shape of languages. In order to get a more detailed view on these interactions the second hypothesis investigates self-assessed singing ability as a sub-component of musicality and shows that people with higher singing skills rate the languages generally as more erotic. Both the standardized effects of singing skills and familiarity can be categorized as significant positive small effects, however the effect of singing skills on eros ratings is bigger than that of familiarity (recognizing but not speaking or having knowledge of the languages), while both must still be considered as small. Interestingly, this is not the case for the instrumentalists—which might bring up the question of whether musicality should be assessed as a general component or split into subparts in future research.

Generally speaking, the primary hypothesis (about the impact of general musicality) has to be rejected. However, a trend that self-perceived musicality in general positively predicts the eros ratings was found. The secondary hypothesis can be accepted. Singing skills have a significant effect on the eros ratings of language—perhaps showing that shared vocal features might explain this interplay. In other words, the relevant component of “musicality” in the present data appears to be vocal musicality. People who sing perceive unfamiliar languages as more erotic. This distinction helps explain why instrumental skills did not show the same association with eros ratings. The result that singing skills are the stronger predictor of affective-aesthetic language ratings, might be explained due to the shared vocal and prosodic features between singing and speech and seeing that they are on the same “vocal” dimension in the Musilinguistic Continuum (Brown, 2022). Especially music with lyrics lies relatively close to spoken language in the continuum between music and language, since both lie on the vocal dimension and use lexical features. In contrast, instrumental music does not rely on lexical features and is therefore behind the linguistic wall (Brown, 2017). The findings of the current study show that singers rate language higher on the eros dimension—which will be discussed in the following sections with two interpretative approaches. On one hand, singers could show higher affective-emotional ratings of languages because of a generally higher vocal aptitude or more intense attraction to voice. On the other hand, seeing that the stimulus sample consisted of “rarer” European languages, they could be the connaisseurs of voice in a sense of having refined taste and apperception for vocal productions / spoken language even of lesser-known languages and in a sense going against the popular opinion (exoticity effect). It must be pointed out that these interpretations must be taken with a grain of salt—seeing that the present analysis relies heavily on self-assessed data.

The first approach argues for the closeness of singing and speaking. As portrayed by the dimensions we can see that these two categories, although often treated as separate, share a common dimension and means of production (the human vocal tract) and are in most parts lexical—considering the dominance of pop music as opposed to non-lexical scat singing. One could argue, especially since self-perceived general musicality showed no significant effect, that it is of importance to discriminate whether a person is an apt “vocalist” or just a musical person in general. In order to further investigate this tentative hypothesis, future research is needed to investigate more dimensions of creativity and might include categories for apt orators or rhetorically trained individuals. This would allow us to compare actors (who vocally perform monologues) to singers. The results of such a study would tentatively play into this general “vocal aptitude” idea assuming greater overlaps between orators/actors and singers than instrumentalists. This “vocal aptitude” framing is not meant to imply that instrumentalists lack aesthetic sensitivity; rather, it proposes that singing is uniquely positioned to train the proprioceptive aspects of voice production that are directly relevant for perceiving speech as embodied action.

Overall, the literature conveys a picture of a general musicality approach but there are studies that investigated differences of these sub-dimensions and how they affect various cognitive domains. Christiner and Reiterer (2015) found that the singers outperform instrumentalists in foreign accent imitation, which argues for a “vocal aptitude” complex. Mansens et al. (2018) found a trend that instrumentalists had a higher cognitive processing speed than singers. However, their distinction was stronger between musicalists and non-musicalists than in comparison of sub-dimensions of musicality, which would argue rather against splitting musicality into sub-dimensions. Joyal et al. (2024) focused on comparing instrumentalists to singers in their study. They found that instrumentalists outperformed singers in terms of accuracy and reaction time. Singers had better accuracy in a working memory task. Even though they discuss the different associations between various musical activities and executive functions, they conclude that there is a general positive relationship between music-making and executive functions. These three studies should highlight the mixed picture that is presented in the literature and more research into the differences and similarities of multiple creative domains might yield new insights into the interplay of perception / cognition and creativity.

### 5.2 Voice connaisseurs, novelty, and rarity

The exploratory analysis aimed to investigate the effects in individual languages and tried to shed light on which languages are more prone to be influenced by musical ear. Significant effects, when controlling for familiarity, were found for Icelandic, Turkish, Norwegian, Greek, Czech, Albanian and Welsh. The first glance over this list of languages already indicates that there might be no clear influence of specific language families. Amongst the stimuli languages, only Portuguese (34% recognition rate) and Greek (32% recognition rate) showed a slightly higher recognition rate, but the others were only modestly recognized (overall mean 14% recognition rate). Thus, except for Portuguese and Greek (generally well-known, but are not amongst the most frequently learned foreign languages of the world), one could say that those do sound “rarer” to the investigated persons. The intention to explore if an effect is bigger for a specific language family seems not fruitful. For example, Icelandic had the biggest effect, but the other Germanic languages (Danish, Norwegian and Swedish) were not in the forefront. The principal idea behind the exploratory analysis—whether languages that are “rarer” because they are less recognized or associated with smaller speaker-communities, including L2 (foreign/second-language) learning communities, and are therefore perceived as more exotic and often more typologically distant (Reiterer et al., 2020; Kogan and Reiterer, 2021)—also seems hard to prove. Seeing that Greek with roughly 12 million L1 speakers worldwide (Worlddata.Info, n.d.) and Polish with roughly 35 million native speakers had the third and fourth highest effect, respectively. Further analysis regarding the phonology and the sound inventories of the language that fared better have to be done in the future. However, it seems that this effect is rather widespread and does not affect individual linguistically defined language families.

The second approach to set the results into a bigger picture focuses on the nature of the stimuli used in the experiment. It remains to be discussed whether there is a general tendency to experience and report eros over all languages, or whether this effect is driven by the sample consisting of rarer languages, in which case the effect may stem from musically inclined individuals being more receptive to unfamiliar sounds or more open to novel experiences (exoticity effect). The sample consists of “rarer” languages, meaning languages with a relatively smaller recognition or set of speakers/second language learners in the European context. Portuguese is an exception on the global level with 196.78 million native speakers in Brazil, and a global total of about 226.78 million people with Portuguese as their mother tongue (Worlddata.Info, n.d.). However, all of the ratings/cases of languages that the participants spoke, even on a very basic level, were excluded from the procedure. Furthermore, the languages that participants recognized, without any active knowledge in the language, were categorized as being familiar and familiarity was controlled by including it in the calculated model (and reporting the small significant effect it has). Keeping all of these measures in mind to exclude the cultural biases that one might have with certain languages—a small significant positive effect of self-assessed singing on affective-aesthetic language ratings on the dimension of eros was still found. Interestingly the effect of singing on affective-aesthetic language ratings was greater than that of familiarity, which usually is considered an important influence in the line of the mere exposure effect (Zajonc, 1968). Even though the effect of singing was greater than the effect of familiarity, they both are considered of small scale. Cui et al. (2015) critically discussed the mere exposure effect and the influence of familiarity on the preference for pitch probability profiles. They conclude that overexposure to the pitch probability profile increases familiarity but decreases preference. Future research could address this nuanced influence of familiarity on perception.

The significant higher eros ratings of rarer languages from singers might point into the direction of singers being “voice connaisseurs”. This, simply put, argues for a refined vocal perception and proprioception which includes kinaesthetic/proprioceptive anticipation and imagination. This includes a sense of awareness and feeling of how one's own articulatory apparatus works. That allows singers more nuanced affective-aesthetic ratings on lesser-known stimuli, because they might have a better developed proprioceptive awareness (embodiment) of their vocal articulatory apparatus. This mechanism or framework in language acquisition and language processing has recently been referred to as “proprioceptive resonance” (Sanna, 2025). Seeing that singing skills had a greater effect on eros ratings than familiarity, it seems that singers are more excitable by languages and their sounds. They still attribute eroticity to some languages, which others find less “thrilling”. The present study only has a weak influence on this theory, seeing that no specific tests for a general sense of embodiment have been performed. Therefore, this claim would need further testing to get a better understanding of whether there is a causal connections between these two features.

Building on this second approach, singers may be more likely to engage in kinaesthetic/proprioceptive anticipation when listening to unfamiliar speech. In this sense, “proprioceptive resonance” (Sanna, 2025) offers a useful conceptual lens: unfamiliar vocal patterns may still elicit embodied simulation of producibility and expressive control, thereby maintaining (or amplifying) affective-approach responses even when semantic familiarity is absent.

### 5.3 Eros in relation to beauty

*Post hoc* exploratory analyses were additionally conducted for beauty. In contrast to eros, beauty did not show a comparably informative pattern and did not add explanatory value to the central theoretical account. This divergence is conceptually plausible. Beauty judgments are often influenced by culturally learned standards and higher-order evaluative processes, whereas eros more directly reflects motivational, approach-related affect that is closer to attraction and mate-choice relevant mechanisms. Within the present design, eros therefore constitutes the more diagnostic outcome for evaluating embodiment-based accounts of phonaesthetic language perception.

Age and gender were examined as exploratory moderators. As these analyses were not originally planned, they are interpreted exploratively. An effect was found for gender indicating that female participants rated the all-female voices of the sample lower than male participants on the eros scale. This could point to a small hetero bias in the sample, which would assume that male participants prefer female voices. This stands in contrast to earlier findings (Reiterer et al., 2020), where female voices were generally rated more favorably. The influences of female voices are, however, not so easily to be put into the wider frame of voice perception which would open up another dozens of possible pathways. Recent work in the field of voice perception (Lozo, 2026) highlights that breathiness and higher pitch index “warm” and “approachable” femininity. Male participants may be more likely to interpret such cues within an attraction-relevant framework. Whereas female participants may process the same cues through social-evaluative filters. This asymmetry could contribute to observed gender differences in the sample. Furthermore, this should highlight how this simple exploratory analysis in this study cannot address the multifaceted influences of voice characteristics and the associations they elicit. Since this was not the main focus of the study, no additional questions regarding the sexuality and sexual preferences of the participants were asked and therefore additional analyses into this topic are not possible at this point.

### 5.4 Limitations

Several limitations qualify the interpretation and generalizability of the findings. First, the stimulus set was restricted to European languages. Although overall recognition rates were low and familiarity was statistically controlled, shared linguistic proximity within Europe may still shape the overall ratings of the languages and might lead to these results not being applicable to all the world's languages. Replications with languages outside the European context are therefore necessary to test the robustness of the observed effects. The focus on European languages was chosen in order to ensure that most of the speakers could come in person to the sound studio and to therefore ensure the high quality of the recordings.

Second, all musicality measures were assessed via self-report using a multi-item scale with good internal consistency (Cronbach's α = 0.80). While the measure appears reliable and non-redundant, self-report cannot substitute for objective indices of perceptual or performance-based ability. The study of Coumel et al. (2019), however, stresses that self-assessment is yielding similar results to more detailed ways of assessing singing skills. This rather simple assessment was chosen for the sake of test efficiency and keeping the maximum experiment time to a limited amount. Future studies would benefit from combining self-report with behavioral markers of vocal-motor and auditory skills.

Third, no sexuality-related variables (e.g., sexual orientation) were collected. Given the conceptual link between eros and attraction, future work could investigate whether these factors moderate eros ratings or relate to individual differences in the affective appraisal of vocal stimuli. Seeing that a small hetero bias was found in the data, with female participants rating female voices lower than male participants.

Fourth, sociolinguistic and cross-cultural biases cannot be fully ruled out. Even with statistical control of familiarity, stereotypes, prestige associations, and ideologies about languages may contribute to evaluations. This, however, seems to be omnipresent when working with languages. Seeing that a language is always inextricably intertwined with cultural identities and markers. Novel approaches in research that focus on the investigation of the imposed norms we associate with languages would be fruitful to address this issue.

Fifth, although voice characteristics were carefully controlled, vocal variation cannot be fully eliminated as a potential influence. Two independent stimulus sets were used, each consisting of professionally recorded, similar-aged female speakers produced under standardized conditions in the University of Vienna MediaLab. Recordings were matched in recording quality, prosodic clarity, and overall vocal timbre to minimize systematic voice differences. Nevertheless, subtle acoustic parameters such as pitch, breathiness, or micro-prosodic variation may still shape listener perception. Given evidence that even small acoustic differences can activate affective associations, future studies could focus on isolating specific vocal parameters. Additionally, the restriction of using younger female speakers, in order to keep the stimuli comparable, might also hinder the generalizability of the results. This renders the question of the influences on cross-gender voice perception unanswered. We suggest that future studies should address this research gap.

## 6 Conclusion

Taken together, the findings of the present study support a notion of vocal skills influencing the perception of vocal productions, like speech. Self-perceived singing skills were the only significant predictor of high eros ratings, implying that the eroticity of a language is specifically tied to the human voice. Participants with higher singing skills rated unknown languages on average higher on the affective-aesthetic dimension of eros / eroticity. The influence of singing skills was even slightly stronger than the “mere-exposure effect” (liking what is familiar), pointing into the direction that a singer's aesthetic judgment is driven by the sound itself rather than cultural recognition. Instrumentalists, on the other hand, did not show this effect, which might lead to an understanding of a shared vocal domain (“vocal aptitude”), as opposed to a broad general musicality. In the literature musicality is often discussed as a separate domain, distinct from language, however, theories on Musilanguage and other recent theories stress the similarities and the possible co-evolution of these two human abilities. The present study supports the continuum between music and language on the vocal dimension and argues for a more nuanced approach to investigate different forms of creativity. Future research might include further sub-dimensions, in order to get a more fine-grained picture on the intertwined influences between creativity and aesthetic judgements.

## Data Availability

The raw data supporting the conclusions of this article will be made available by the authors, without undue reservation.
